# Draft Genome Sequence of the Nitrogen-Fixing *Rhizobium sullae* Type Strain IS123^T^ Focusing on the Key Genes for Symbiosis with its Host *Hedysarum coronarium* L.

**DOI:** 10.3389/fmicb.2017.01348

**Published:** 2017-07-26

**Authors:** Gaurav Sablok, Riccardo Rosselli, Torsten Seeman, Robin van Velzen, Elisa Polone, Alessio Giacomini, Nicola La Porta, Rene Geurts, Rosella Muresu, Andrea Squartini

**Affiliations:** ^1^Department of Biodiversity and Molecular Ecology, Research and Innovation Centre, Fondazione Edmund Mach San Michele all'Adige, Italy; ^2^Division de Microbiología, Universidad Miguel Hernandez San Juan de Alicante, Spain; ^3^Victorian Bioinformatics Consortium, Monash University Melbourne, VIC, Australia; ^4^Laboratory of Molecular Biology, Department of Plant Science, Wageningen University Wageningen, Netherlands; ^5^Department of Agronomy, Food, Natural Resources, Animals and Environment, University of Padova Legnaro, Italy; ^6^Department of Sustainable Agrobiosystems and Bioresources, Research and Innovation Centre, Fondazione Edmund Mach San Michele all'Adige, Italy; ^7^MOUNTFOR Project Centre, European Forest Institute San Michele all'Adige, Italy; ^8^Institute of Animal Production Systems in Mediterranean Environments-National Research Council Sassari, Italy

**Keywords:** *Rhizobium sullae*, type strain, *Hedysarum coronarium*, *Sulla coronaria*, nod genes, host-specific symbiotic adaptation, root-nodule bacteria, nitrogen fixation

## Abstract

The prominent feature of rhizobia is their molecular dialogue with plant hosts. Such interaction is enabled by the presence of a series of symbiotic genes encoding for the synthesis and export of signals triggering organogenetic and physiological responses in the plant. The genome of the *Rhizobium sullae* type strain IS123^T^ nodulating the legume *Hedysarum coronarium*, was sequenced and resulted in 317 scaffolds for a total assembled size of 7,889,576 bp. Its features were compared with those of genomes from rhizobia representing an increasing gradient of taxonomical distance, from a conspecific isolate (*Rhizobium sullae* WSM1592), to two congeneric cases (*Rhizobium leguminosarum* bv. *viciae* and *Rhizobium etli*) and up to different genera within the legume-nodulating taxa. The host plant is of agricultural importance, but, unlike the majority of other domesticated plant species, it is able to survive quite well in the wild. Data showed that that the type strain of *R. sullae*, isolated from a wild host specimen, is endowed with a richer array of symbiotic genes in comparison to other strains, species or genera of rhizobia that were rescued from domesticated plant ecotypes. The analysis revealed that the bacterium by itself is incapable of surviving in the extreme conditions that its host plant can tolerate. When exposed to drought or alkaline condition, the bacterium depends on its host to survive. Data are consistent with the view of the plant phenotype as the primary factor enabling symbiotic nitrogen fixing bacteria to survive in otherwise limiting environments.

## Introduction

The interaction between rhizobia and legumes in root nodules is an essential element in sustainable agriculture, as this symbiotic association is able to enhance biological fixation of atmospheric nitrogen (N_2_), and is also a paradigm in plant–microbe signaling (Young et al., 2006; Giraud et al., 2007; Wang et al., 2012). Knowledge of the whole genome would allow the specific features of each rhizobium to be identified. The prominent feature of this group of bacteria is their molecular dialogue with plant hosts, an interaction that is enabled by the presence of a series of symbiotic genes encoding for the synthesis and export of signals triggering organogenetic and physiological responses in the plant (Spaink et al., 1987; Long, 2001). In recent years, significant progress has been made in resolving the complex exchange of signals responsible for nodulation through genome assembly, mutational, and expression analysis, and proteome characterization of legumes (e.g., Sato et al., 2008; Young et al., 2011; Marx et al., 2016) and rhizobia (e.g., Giraud et al., 2007; Tolin et al., 2013; Čuklina et al., 2016; Remigi et al., 2016). In a previous study (Squartini et al., 2002), we described a novel species, *R. sullae*, that specifically induces symbiotic nodulation in the legume sulla (*Hedysarum coronarium* L. syn. *Sulla coronaria* [L.] Medik.; Faboideae; Hedysareae). We had previously provided the first description of the infection process of this legume by its bacterial symbiont and their morphological peculiarities (Squartini et al., 1993). Sulla is found in the Mediterranean basin with a distribution from northern Africa to southern Spain and southern Italy. It is of particular importance in agriculture due to its ability to adapt to drought and coastal conditions (Douglas, 1984), and is therefore an ideal subject for studying salt tolerance (range limit 150–700 mM NaCl), alkaline tolerance (up to pH 9–10.5), and drought stress (ranging from 0.5 to −0.95 MPa for PEG; Fitouri et al., 2012; Issolah et al., 2012). Biochemical and genetic characterization of several bacterial strains nodulating sulla (Struffi et al., 1988; Muresu et al., 2005) allowed us to select *R. sullae* isolate IS123^T^ (= USDA 4950^T^ = DSM 14623^T^) as type strain. Phylogenetic analyses (Squartini et al., 2002) suggest it is closely related to the widely-studied congeneric *Rhizobium leguminosarum* (symbiont of peas) and *Rhizobium etli* (symbiont of beans).

Focusing, in particular, on the genes ruling the symbiotic association with the host plant, we sequenced the genome of the *R. sullae* type strain IS123^T^ in order to: (1) compare this genome with other members of the order Rhizobiales, including a conspecific isolate (*R. sullae* WSM1592, Yates et al., 2015), two congeneric cases (*R. leguminosarum* bv. viciae and *R. etli*), and various genera within the legume-nodulating taxa; (2) determine whether or not the type strain of *R. sullae*, which comes from a plant that still grows in the wild, carries a richer array of genes for the symbiotic interaction with its host; (3) assess whether or not the traits allowing the host plant to endure extreme soil conditions (drought and alkalinity) are also mirrored by appropriate determinants in the bacterial genome.

## Materials and methods

The *R. sullae* strain IS123^T^ has been previously described as a new species by Squartini et al. (2002). Genomic DNA was isolated from exponentially-growing liquid cultures in Yeast-Mannitol broth. Cultures were lysed and washed, the DNA was extracted using chloroform/phenol, and, after ethanol precipitation, it was purified using a Qiagen DNeasy blood and tissue kit, according to the manufacturer's protocol (Qiagen, Hilden, Germany). Libraries were prepared with the TruSeq DNA Library Preparation Kit (Illumina Inc.), as described by the manufacturer. DNA was sequenced using the Illumina HiSeq platform, and the sequences assembled with an Edena (Exact DE Novo Assembler; Hernandez et al., 2008), which uses an overlap layout consensus algorithm with an overlap cutoff of 47 bases. Assembled contigs were further scaffolded using SSPACE Basic (version 2.0; Boetzer et al., 2011). The scaffolded version of the assembled genome was used for gene prediction and annotation using two independent pipelines: RAST, available at: http://rast.nmpdr.org/ (Aziz et al., 2008), and the Prokka bacterial genome annotation tool (Seemann, 2014), which uses Prodigal (Hyatt et al., 2010) for prokaryotic gene identification. While running the Prokka, the Rhizobiales order was selected to represent the genomes from this class in order to increase the robustness of the annotations. The Prokka and RAST outputs revealed slight differences in the functional annotations, so we manually checked them focusing on the symbiotic nodulation and nitrogen fixation genes. The curated version of Prokka was used as the final annotation of *R. sullae*. To ascertain the similarity between the assembled genome and previously published genomes of *Rhizobium etli* and *R. sullae* WSM1592, we estimated average nucleotide identity (ANI), as previously described by Goris et al. (2007). The ANI analysis was limited to the taxa which were expected to share most identities with the strain under study.

A whole-genome orthology search to identify conserved functions across different organisms was run on six additional rhizobial genomes using the Reciprocal BEST BLAST HIT (RBBH) (Ward and Moreno-Hagelsieb, 2014) with an *E*-value cutoff of 1E-5. Genes with identity >60% and coverage higher than 60% were considered orthologous.

The MAPLE resource (Metabolic And Physiological potentiaL Evaluator; available at: http://www.genome.jp/tools/maple/help.html) was used to estimate function abundance and evaluate metabolic and physiological potential. The reference database was KEGG (Kyoto Encyclopedia of Genes and Genomes), and *R. sullae* proteins were mapped and normalized on the ribosomal proteins counts in its pathway database. KAAS (KEGG Automatic Annotation Server) was used for ortholog assignment (KO, Kegg Orthology) and pathway mapping. The PHASTER tool was used to search for sequences of phages and prophages (Arndt et al., 2016). To ascertain whether any genes relevant for rhizobia could be missing, an HMM (Hidden Markow Model) search of the Rhizobiales was carried out to extract the core pan-genome, which was aligned with the genomes of the other rhizobia and scored for conservation percentage. The analysis was carried out using bcgTree, which is a bacterial core gene analyzer (http://www.dna-analytics.biozentrum.uni-wuerzburg.de, Ankenbrand and Keller, 2016).

## Results

17,902,513 paired end (PE) reads (2 × 100 bp) were obtained, accounting for a total of 9.1 Gb sequence information.

The draft genome assembly contains 447 contigs with a total assembly size of 7,889,725 bp and an N50 of 72.64 kb, and 317 scaffolds with a total assembly size of 7,889,576 bp and an N50 of 118.24 kb. The longest contig was 296,399 bp, the longest scaffold 488 bp. The whole genome GC content was 59.88% (Table 1). Sequence annotation revealed 7,776 protein coding sequences (CDSs), which is more than those found in the other *R. sullae, R. leguminosarum*, and *R. etli* genomes reported so far. A total of 51 tRNA genes and 6 rRNA (rrn) operons were identified.

**Table 1:** Comparative genome features of *Rhizobium sullae* IS123^T^, its co-specific *R. sullae* WSM1592, and its two taxonomically closest relatives *R. leguminosarum* biovar *viciae* 3841 and *R. etli* CFN42.

**Features**	***R. sullae* IS123^T^**	***R. sullae* WSM1592**	***R. leguminosarum* bv. *viciae* 3841**	***R. etli* CFN42**
Genome (bp)	7,889,576	7,530,820	7,751,309	6,530,228
GC average %	59.88%	59.87	60.86%	60.54
rRNA operons	6	5	3	3
tRNA	51	47	52	50
Total CDS	7,776	7,453	7,265	6,034

The raw reads of the *R. sullae* IS123^T^ genome are available in the European Bioinformatics Institute (EBI) database under project number PRJEB9435. The genome assembly can be accessed under the code ERZ403196 (Sample id: ERS738956, Assembly accession: GCA_900169785, WGS account: FWER01; Scaffold accession range: FWER01000001-FWER01000317).

The gene predictions, CDS FASTA files, and the corresponding proteins are all provided as Supplementary Datasets 2–6.

**Figure 1:**
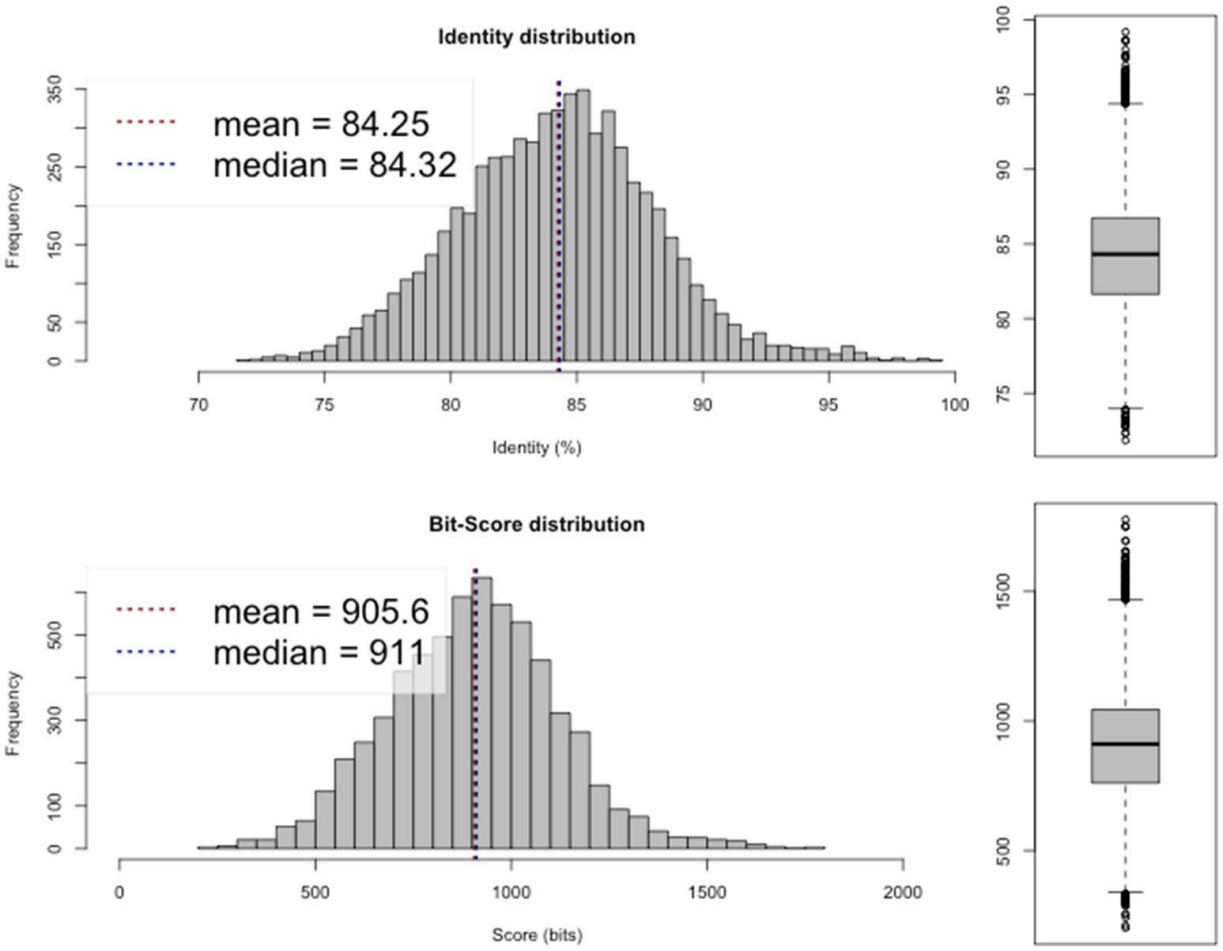
Average Nucleotide Identity (ANI) plot between *R. sullae* IS123^T^ and *R. etli* CFN42.

**Figure 2:**
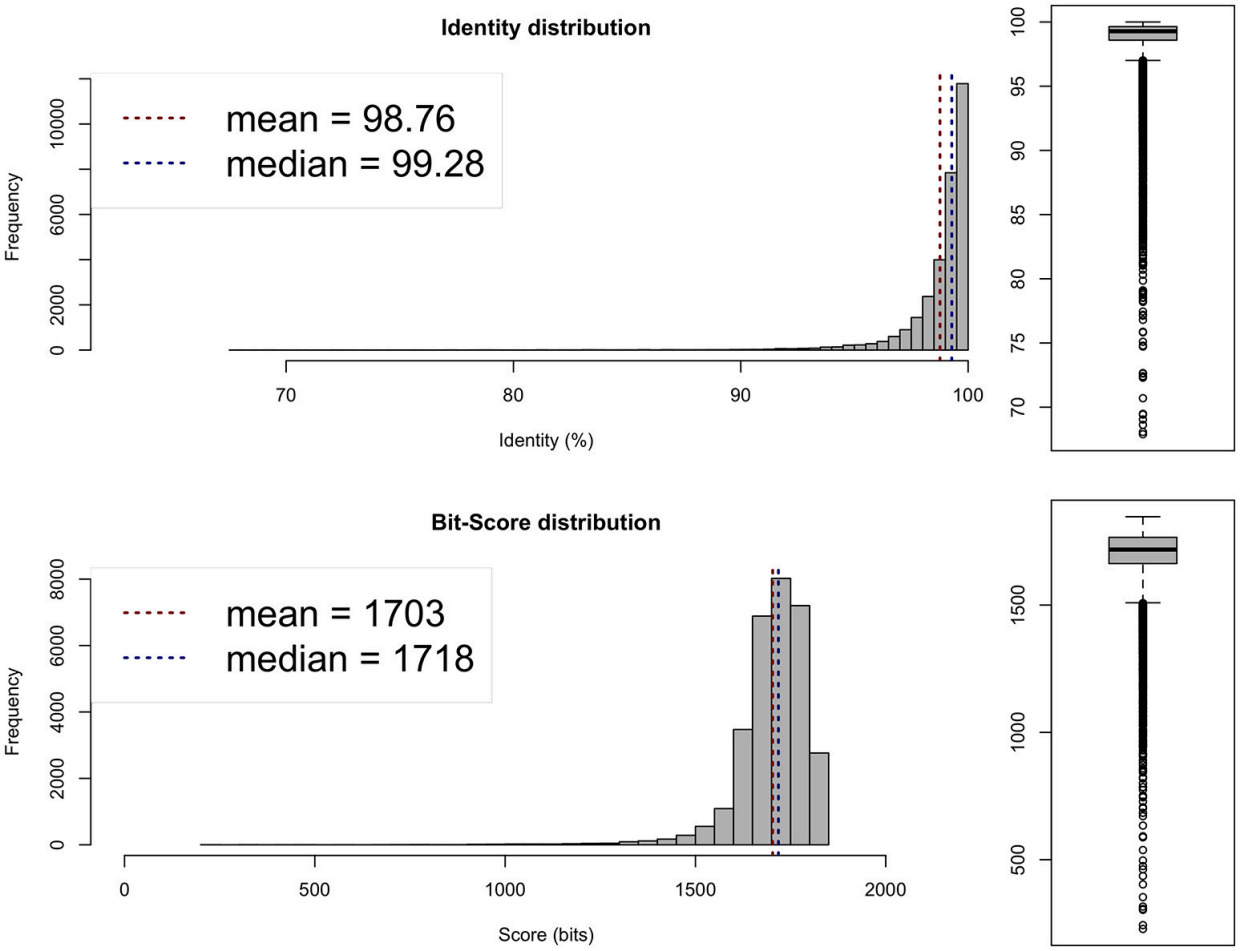
Average Nucleotide Identity (ANI) plot between *R. sullae* IS123^T^ and *R.sullae* WSM1592.

Average nucleotide identity (ANI; Goris et al., 2007; available from http://enve-omics.ce.gatech.edu/ani/) was calculated to assess the similarity between the assembled genome and the genomes of *R. sullae* WSM1592 and *R. etli* CFN42. The analysis revealed a high average ANI (97–98%) between the sequenced genome and the genomes of the two afore-mentioned related species (Figures 1, 2), confirming their evolutionary closeness.

In addition, the *R. sullae* IS123^T^ proteins were mapped against the KEGG pathways database, and the result normalized by the ribosomal proteins. The resulting KEGG orthology assignment of genes and modules, and the corresponding rarefaction curves are shown in Supplementary Material S1, along with an annotated map of a prophage that was found in the *R. sullae* IS123^T^ genome.

Core pan-genome analysis of the Rhizobiales was carried out to see whether any major genes were missing. The core was found to include 108 genes and the analysis indicated that the *R. sullae* IS123^T^ genome presented here is more than 99% complete with respect to the core rhizobial pan-genome. This confirms both the trustworthiness of the ortholog analysis and the overall good quality of the genome coverage. The results of the pan-genome analysis are available as Supplementary Dataset 7.

**Figure 3:**
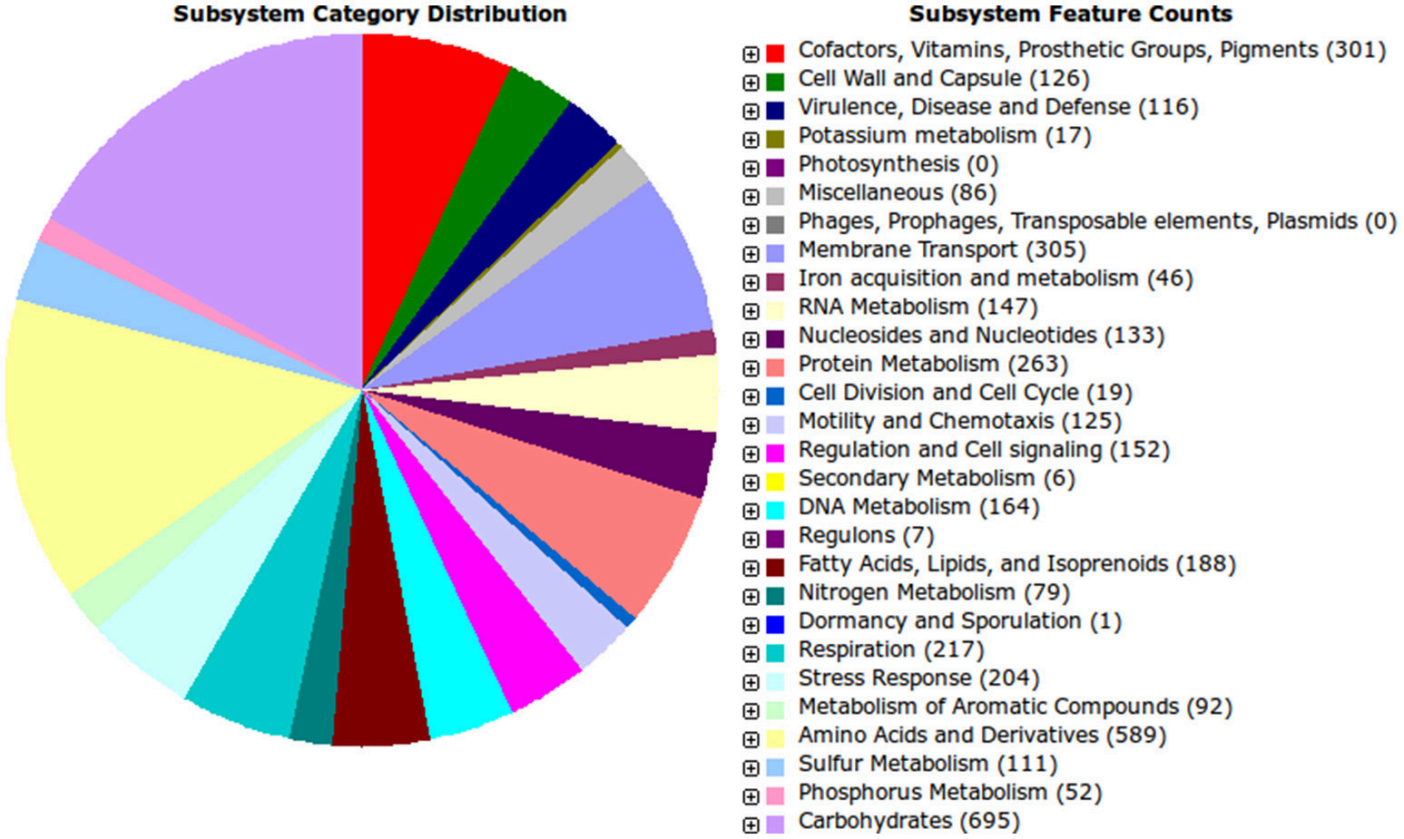
Gene category classification results. The annotation data obtained from the RAST procedure were used to assemble the image.

Functional categorization of the genes present in the IS123^T^ strain based on the RAST annotation is shown in Figure 3. While this kind of analysis is often not exhaustive, as the output depends on the accuracy of the gene classification databases, a general picture can be drawn. The majority of its genes are predicted to be involved in carbohydrate metabolism, which is consistent with the rhizobial lifestyle within which exopolysaccharides are important traits involved in host plant recognition, lectin binding, and attachment to root surfaces. In addition, a considerable array of genes relate to membrane transport, and their involvement in the secretion machinery for metabolites and signals is also important in plant–microbe interactions.

Sequence annotation confirmed the presence of genes involved in nodulation (*nod*) and symbiotic N_2_ fixation (*nif* and *fix*). The *R. sullae* type strain genome displayed all the essential nodulation genes, which are required for early symbiotic nodulation via nod factor production (Baev et al., 1992; Young et al., 2006). Among the nod genes, *nodA* was present in two copies, the first a short truncated form located in the *nodABC* operon, the second a supposedly functional full copy upstream of a gene for carbonic anhydrase. Opposite the truncated *nodA* and its nod box (the NodD regulator-binding cassette for nod genes) in the *nodABC* operon on the complementary strand, there is a copy of *nodD* interrupted by the insertion element ISRh1 (Meneghetti et al., 1996). Two other intact copies of *nodD* are located elsewhere, one of them close to its cognate receptor *syrM*, while *nodB* and *nodC* are present in the afore-mentioned operon, and *nodH* is immediately downstream of *nodC*. The nod factor export machinery also appears to be in place, due to the presence of the transport-related *nodIJ* operon, whose origin has been traced from Beta- to Alpha-Proteobacteria (Aoki et al., 2013). The presence of other defined nod genes means we can predict the following potential characteristics of the corresponding nod factors: regular acylation (presence of *nodFE*), O-acetylation (*nodL* and *nodX*), sulfation (*nodH, nodPQ*), and a putative O-carbamoylation (*nolO*). These features, encoding for precise nod factor decorations, may account for the very tight host specificity of this rhizobium for its symbiont plant *H. coronarium*.

Among the *nif* genes, which are involved in nitrogen fixation, we identified the structural nitrogenase units *nifH, nifD*, and *nifK*, plus regulatory and accessory determinants (*nifA, nifB, nifE, nifN, nifS, nifT, nifU, nifW, nifZ*, and the 4Fe-4S ferredoxin nitrogenase-associated gene). Among the fix genes, we detected *fixL, fixS*, and *fixC*, which potentially encode a symbiotically essential cbb_3_ high-affinity terminal oxidase, a rate-limiting component for symbiotic N_2_ fixation (Patschkowski et al., 1996). In fact, *ab-initio* gene prediction using Prodigal identified three copies of the cbb_3_ high-affinity terminal oxidase in this *R. sullae* IS123^T^ genome. In confirmation of the presence of other genes related to symbiosis, we also detected *sufBCDE*, the gene cluster acting as house-keeping storage genes of FeS clusters (Trotter et al., 2009). Furthermore, we found two copies of *gndA* gene, which catalyzes 6-phosphogluconate dehydrogenase and is the main source of catabolizing sugars in rhizobial species using the Entner-Doudoroff pathway, as previously reported for *R. leguminosarum* and *R. etli* (González et al., 2006; Young et al., 2006).

In order to assess the uniqueness of the genes and the level of conservation of those shared across related taxa, a whole genome orthology search was run on six additional rhizobial genomes. Besides *R. sullae* IS123^T^, we used the following species for the analysis (gene counts are indicated in parenthesis): *R. sullae* WSM1592 (6985), *R. leguminosarum* bv. *viciae* 3841 (7281), *R. etli* CFN42 (5963), *Sinorhizobium meliloti* 1021 (6217), *Mesorhizobium loti* MAFF303099 (7283), and *Bradyrhizobium japonicum* USDA110 (8317).

Occurrences of the corresponding orthologous genes are shown in the “Supplementary Dataset 1, Orthology analysis.” A zero (0) in a cell indicates the absence of an orthologous gene. The second worksheet contains the gene nomenclature and features of all of the genes compared.

On inspecting all the genes known to correspond to symbiotic traits, (nodulation, nitrogen fixation, or their ancillary metabolism), we found that, compared with the type strain *R. sullae* IS123^T^, its conspecific *R. sullae* WSM1592 lacks *nifQ* (the molybdenum donor for nitrogenase synthesis), the second copy of the structural *nifH* gene for nitrogenase, a copy of the *fixN* Cytochrome c oxidase subunit, a copy of the *fixL* oxygen sensor protein, a copy of the *fixK* nitrogen fixation regulation protein, and the gene for ferredoxin I, an enzyme linked to the electron flux for N_2_ reduction to nitrogenase.

The symbiotic genes that are present in *R. sullae* IS123^T^ but have no orthologs in either of the two species of the same genus and closest to *R. sullae* according to 16S rRNA taxonomy, namely *R. leguminosarum* bv. *viciae* and *R. etli*, include, in addition to the above: the *nodG* (*fabG*) 3-oxoacyl acyl-carrier protein reductase; *nodM*, encoding functions for efficient Nod signal production; *fixJ*, the response regulator inducing nif operons in response to microaerobiosis within the nodule tissue; the putative nitrogen fixation protein gene *fixT*; a copy of the nod factor synthase *nodA*; a copy of the *nodD2* and *syrM* genes encoding for flavonoid-responsive transcriptional activators; and a copy of the *nodU* putative carbamoyl transferase.

Interestingly, the ortholog search across the seven taxa, including the three different genera *Bradyrhizobium, Mesorhizobium*, and *Sinorhizobium* (*Ensifer*), revealed the *R. sullae* IS123^T^ genome to be the one displaying the highest number of genes related to symbiosis, which was 59.

Since the host plant *H. coronarium*, as mentioned in the introduction, has remarkable properties of tolerance to several environmental stress factors, such as salinity, drought, and alkaline soil pH, and displays regular root nodulation under such conditions, we inspected the genome of its *R. sullae* microsymbiont to search for genes which could account for corresponding genotypes on the bacterial side.

We identified genes important in maintaining osmoregularity, which included the osmotically inducible protein C. However, in bacteria this gene is reported to be related not to saline conditions, but rather to responses to organic hydroperoxides and reactive oxygen species in general (Lesniak et al., 2002).

An integral membrane protein, YggT, involved in response to extracytoplasmic stress (osmotic shock) is present, but this gene appears to be widespread among bacteria, including *Escherichia coli*, and is not characteristic of isolates endowed with particular tolerances. The same goes for Aquaporin Z, relevant for osmoregulation but present in *E. coli* as well (Delamarche et al., 1999). In general, the *R. sullae* genome does not reveal its distinctiveness in terms of membrane physiology and selectivity, and it contains customary features, such as the osmolarity sensor *envZ*, the osmosensitive K+ channel histidine kinase KdpD, and the beta-(1–>2) glucan export ATP-binding/permease protein NdvA, which is responsible for the synthesis of osmoregulated periplasmic glucans. These oligosaccharides are common not only to most rhizobia but to the whole Proteobacteria phylum, in which glucan concentration in the periplasm increases in response to a decrease in environmental osmolarity (Bohin, 2000). This finding is again not indicative of any adaptation to salty conditions as the response is instead to the opposite scenario (diluted circulating solution). The presence of this gene is nevertheless worth remarking on, as it is another corollary to the symbiotic proficiency of *R. sullae*. Indeed, mutations in the same *ndvA* gene in *S. meliloti* result in the delayed formation of numerous small white nodules that are not invaded by the mutant bacteria and are consequently unable to fix nitrogen (Ielpi et al., 1990).

Considering that the host plant is able to withstand 700 mM NaCl, and is regularly encountered with well-developed root nodules in the Tuscan pliocenic clays at pH 9.6, the absence of prominent traits of extremophily among the genes of its bacterial symbiont could appear to be in contrast to the habitats where the host plant and bacteria meet. However, it is interesting to recall that in a prior study on the stress tolerance of several strains of *R. sullae*, including the present type strain IS123^T^, compared with other rhizobia (Struffi et al., 1988), the *R. sullae* strains did not perform any better than the other rhizobia. Regarding NaCl, in most strains growth was limited to 0.5%, the same limit in *R. leguminosarum* bv. *trifolii* (clover symbiont) and *R. leguminosarum* bv. *viciae* (pea symbiont). The limit was 1% in only two strains, but this was still lower than in *S. meliloti* (symbiont of alfalfa), which tolerated values up to 3%. As for alkali tolerance, *R. sullae* did not perform very differently from the others, and *R. sullae* IS123^T^, in particular, was able resist up to pH 8.5, the same level attained by the pea and clover symbionts, and lower than that recorded for the alfalfa symbiont (Struffi et al., 1988). These data are consistent with the lack of obvious stress tolerance traits in *R. sullae* emerging from the present genome analysis, and allow for an interpretation focused on the ecological coexistence of tolerant hosts and non-tolerant microorganisms. In our earlier experiment with *R. leguminosarum* bv. *trifolii* (Casella et al., 1988), we demonstrated that the ability to survive in the presence of increasing doses of different heavy metals was dependent on the plant's phenotype, irrespective of the bacterial phenotype. For example, at chromium levels which were still tolerated by clover but high above the minimum inhibitory concentration for the rhizobium, the latter was nevertheless able to effectively nodulate the plant. Interestingly, we also noticed that on curing those rhizobia of their large symbiotic plasmids, they lost the ability to induce and invade root nodules, but at the same time their intrinsic level of chromium tolerance increased. These data, along with the afore-mentioned absence of either genomic or phenotypic evidence for salt- or alkali-tolerance traits in *R. sullae*, lead us to the view that in these endophytic plant–microbe interactions, of the two partners the plant host is the one that is critically endowed with the ability to occupy challenging environments. In this sense, the symbiosis assumes the role of a shelter for the hosted microorganisms, which could otherwise not endure the same stress factors when facing them as free living cells. In the case of heavy metals, as the study cited showed, it was also rather revealing to see that rhizobia could “opt” between entering the plant, which offers a shielded niche, or renounce the whole symbiotic relationship by dropping an extrachromosomal replicon. The consequent loss of interactivity, due to the absence of the plasmid-borne genes, accounts for several membrane permeability changes, which are related to overall increased resistance to extracytoplasmic stress factors.

In the particular case of *R. sullae*, it should be added that its host, *H. coronarium*, has unique features of environmental adaptation, which, as far as is known, have not been encountered in any other plant. As we described in an earlier report (Tola et al., 2009), this genus is capable of forming modified lateral roots (called shovel roots) that accumulate calcium crystals, a mechanism that explains the plant's ability to grow, often as a unique vegetation form, in calcareous soils of extreme alkalinity. The scavenging of Ca^2+^ ions locally affects the chemical equilibrium of the soil carbonate buffer and allows efficient acidification of the rhizosphere, even in limestone-rich, highly basic soils. In the same report, we showed that *H. coronarium* is able to change the pH of its surrounding solution, even from alkalinity to acidity, while other legumes are only able to exert the reverse effect. These findings further support the evidence that it is the plant's task to withstand the harsh conditions characterizing its habitat. At the same time, the plant is able to improve the conditions in the root microenvironment, allowing its microbial partner to multiply in a situation it would otherwise not be able to withstand, and eventually to be fully rescued within the endophytic and symbiotic domains. The picture of this interaction is thus reconciled with the genome annotations of *R. sullae*, which, as mentioned above, featured genes that were not very different from those of most average bacteria in terms of coping with environmental stress, yet also featured a plethora of genes for symbiotic interactions.

**Figure 4:**
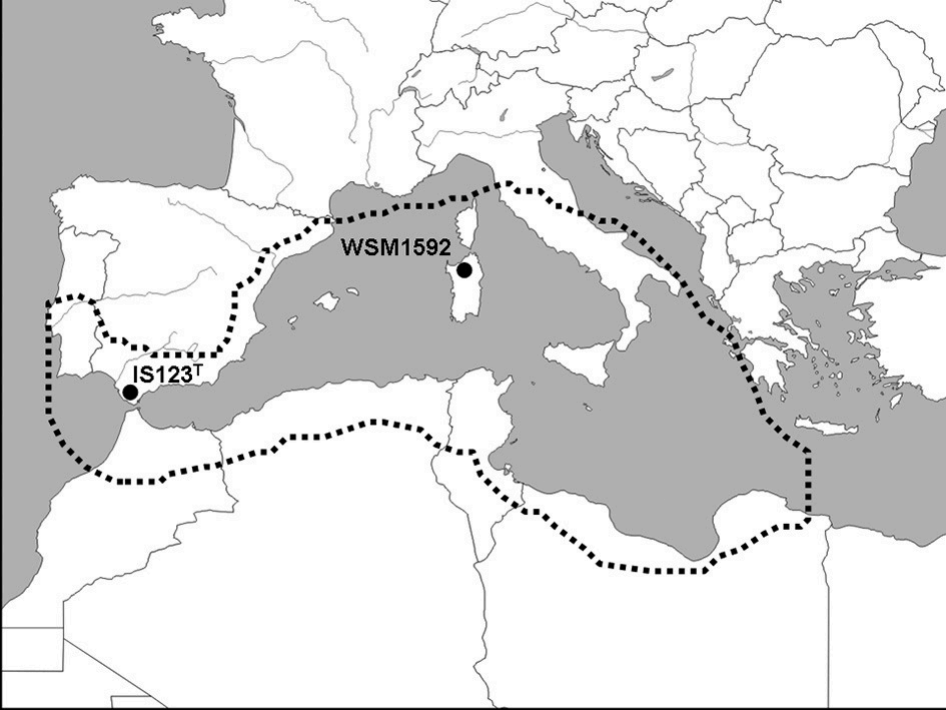
Map of the Western Mediterranean region showing the isolation sites of *Rhizobium sullae* IS123^T^ and WSM1592. The dotted line encompasses sites where the host plant *Hedysarum coronarium* L. (*Sulla coronaria* Medik) is currently distributed, and includes areas where the plant has been attributed the status “native” (Algeria, Morocco, Tunisia, Spain), or “uncertain origin,” i.e., possibly introduced (Corsica, Sardinia, Continental Italy, Sicily, Lybia, Egypt). The plant is also naturalized or cropped in other areas, where it has been intentionally introduced, including the Balearic Islands, Portugal, former Yugoslavia, Malta, Lebanon, and Syria. Source of the distribution data: International Legume Database and Information Service (http://www.legumes-online.net/ildis/aweb/database.htm).

The genome of *R. sullae* IS123^T^ presented here draws attention to the way it differs from the available genome of the cognate strain *R. sullae* WSM1592, and has shown how the type strain appears to be endowed with a richer array of genes pertaining to the symbiotic phenotypes of nodulation and nitrogen fixation. Moreover, there are certain ecological aspects that allow us to make further inferences. These concern the migratory path of the host plant on its colonization route into Europe from the North African plains, which is presumed to coincide with its domestication and subsequent cropping. The type strain sequenced here was, in fact, isolated in the early seventies from a wild stand of its host in Cadiz province, the southernmost part of Spain, facing the Straight of Gibraltar and Africa. Strain WSM1592, instead, was recovered in 1995 from cultivated Sulla plants grown at the Ottava experimental station on the Italian island of Sardinia. The sites where the two strains were isolated within their host home range are shown on the map of the western Mediterranean area in Figure 4. Some geographical as well as ecological variables may account for the observed inter-strain variations between IS123^T^ and WSM1592. In this regard, it is worth remarking that *H. coronarium* is one of the few plant species which still exist in both wild and cultivated conditions. This unique feature makes it possible to sample and compare the genomes of two strains, one from the wild (IS123^T^), the other from an agricultural context (WSM1592), and to investigate the subtle ways in which they differ in spite of an overall conserved genome. The host plant is considered to be native to Algeria, Tunisia and Morocco, as well as Spain (http://www.ildis.org/), where it is found essentially in the southernmost region. The presumed origin of the plant species in the north-western African belt above the Saharan Atlas range is supported by the high frequency of sites in which *H. coronarium* is encountered in natural populations in that area compared with European countries, and by the fact that the related species *Hedysarum flexuosum* is the only legume nodulated by rhizobia, whose 16S rRNA sequence displays a >99% similarity with the *R. sullae* type strain IS123^T^ (Aliliche et al., 2016). These nodules are ineffective, as *R. sullae* is, as far as is currently known, fully symbiotic only with *H. coronarium*.

In conclusion, the present study enabled us to verify ecological correspondences between host–plant lifestyles and bacterial symbiont genotypes. In addition to the interest in comparing genomic features of isolates from cultivated vs. wild Sulla, the present genome, a strain collected from a wild specimen thriving in an arid region of the Mediterranean, provided us with the possibility to investigate, and to rule out, the presence of genes related to drought and salt tolerance, which are two major characteristics of the naturally-occurring ecotypes of its plant host. Its range extends throughout the near-desert belt in Northern Africa, and its invasion of Mediterranean Europe has apparently followed a route via Gibraltar, the site where *R. sullae* IS123^T^ was isolated and the transcontinental crossing point.

## Author contributions

AS and RM: conceived the project. Rv, RG, AG, and EP: performed the sequencing GS, TS, NL, and RR: were responsible of bioinformatics analysis. AS: wrote the manuscript.

### Conflict of interest statement

The authors declare that the research was conducted in the absence of any commercial or financial relationships that could be construed as a potential conflict of interest.

## References

[B1] Aliliche K. Beghalem H. Landoulsi A. Chriki A. (2016). Molecular phylogenetic analysis of *Rhizobium sullae* isolated from Algerian *Hedysarum flexuosum*. Antonie Van Leeuwenhoek 109, 897–906. 10.1007/s10482-016-0688-3 27034287

[B2] Ankenbrand M. J. Keller A. (2016). bcgTree: automatized phylogenetic tree building from bacterial core genomes. Genome 59, 783–791. 10.1139/gen-2015-0175 27603265

[B3] Aoki S. Ito M. Iwasaki W. (2013). From β-to α-proteobacteria: the origin and evolution of rhizobial nodulation genes. nodIJ. Mol. Biol. Evol. 30, 2494–2508. 10.1093/molbev/mst153 24030554

[B4] Arndt D. Grant J. Marcu A. Sajed T. Pon A. Liang Y.. (2016). PHASTER: a better, faster version of the PHAST phage search tool. Nucleic Acids Res. 44, W16–W21. 10.1093/nar/gkw387 27141966 PMC4987931

[B5] Aziz R. K. Bartels D. Best A. A. DeJongh M. Disz T. Edwards R. A.. (2008). The RAST Server: rapid annotations using subsystems technology. BMC Genomics 9:75. 10.1186/1471-2164-9-75 18261238 PMC2265698

[B6] Baev N. Schultze M. Barlier I. Ha D. C. Virelizier H. Kondorosi E.. (1992). Rhizobium nodM and nodN genes are common nod genes: nodM encodes functions for efficiency of nod signal production and bacteroid maturation. J. Bacteriol. 174, 7555–7565. 10.1128/jb.174.23.7555-7565.1992 1447128 PMC207465

[B7] Boetzer M. Henkel C. V. Jansen H. J. Butler D. Pirovano W. (2011). Scaffolding pre-assembled contigs using SSPACE. Bioinformatics 27, 578–579. 10.1093/bioinformatics/btq683 21149342

[B8] Bohin J.-P. (2000). Osmoregulated periplasmic glucans in Proteobacteria. FEMS Microbiol. Lett. 186, 11–19. 10.1111/j.1574-6968.2000.tb09075.x 10779706

[B9] Casella S. Frassinetti S. Lupi F. Squartini A. (1988). Effect of cadmium, chromium and copper on symbiotic and free-living *Rhizobium leguminosarum* biovar trifolii. FEMS Microbiol. Lett. 49, 343–347. 10.1111/j.1574-6968.1988.tb02754.x

[B10] Čuklina J. Hahn J. Imakaev M. Omasits U. Förstner K. U. Ljubimov N.. (2016). Genome-wide transcription start site mapping of *Bradyrhizobium japonicum* grown free-living or in symbiosis - a rich resource to identify new transcripts, proteins and to study gene regulation. BMC Genomics 17:302. 10.1186/s12864-016-2602-9 27107716 PMC4842269

[B11] Delamarche C. Thomas D. Rolland J.-P. Froger A. Gouranton J. Svelto M.. (1999). Visualization of AqpZ-mediated water permeability in *Escherichia coli* by cryoelectron microscopy. J. Bacteriol. 181, 4193–4197. 10400575 10.1128/jb.181.14.4193-4197.1999 PMC93919

[B12] Douglas G. B. (1984). Seed production of sulla: a plant for soil conservation. Proc. N.Z. Grassland Assoc. 45, 239–242.

[B13] Fitouri S. D. Trabelsi D. Saïdi S. Zribi K. Jeddi F. B. Mhamdi R. (2012). Diversity of rhizobia nodulating *sulla* (*Hedysarum coronarium* L.) and selection of inoculant strains for semi-arid Tunisia. Ann. Microbiol. 62, 77–84. 10.1007/s13213-011-0229-2

[B14] Giraud E. Moulin L. Vallenet D. Barbe V. Cytryn E. Avarre J. C.. (2007). Legumes symbioses: absence of Nod genes in photosynthetic bradyrhizobia. Science 316, 1307–1312. 10.1126/science.1139548 17540897

[B15] González V. Santamaría R. I. Bustos P. Hernández-González I. Medrano-Soto A. Moreno-Hagelsieb G.. (2006). The partitioned *Rhizobium etli* genome: genetic and metabolic redundancy in seven interacting replicons. Proc. Natl. Acad. Sci. U.S.A. 103, 3834–3839. 10.1073/pnas.0508502103 16505379 PMC1383491

[B16] Goris J. Konstantinidis K. T. Klappenbach J. A. Coenye T. Vandamme P. Tiedje J. M. (2007). DNA-DNA hybridization values and their relationship to whole-genome sequence similarities. Int. J. Syst. Evol. Microbiol. 57, 81–91. 10.1099/ijs.0.64483-0 17220447

[B17] Hernandez D. François P. Farinelli L. Osterâs M. Schrenzel J. (2008). *De novo* bacterial genome sequencing: millions of very short reads assembled on a desktop computer. Genome Res. 18, 802–809. 10.1101/gr.072033.107 18332092 PMC2336802

[B18] Hyatt D. Chen G. L. Locascio P. F. Land M. L. Larimer F. W. Hauser L. J. (2010). Prodigal: prokaryotic gene recognition and translation initiation site identification. BMC Bioinformatics 11:119. 10.1186/1471-2105-11-119 20211023 PMC2848648

[B19] Ielpi L. Dylan T. Ditta G. S. Helinski D. R. Stanfield S. W. (1990). The ndvB locus of *Rhizobium meliloti* encodes a 319-kda protein involved in the production of beta-(1-2)-glucan. J. Biol. Chem. 15, 2843–2851. 2154461

[B20] Issolah R. Tahar A. Derbal N. Zidoun F. Ait Meziane M. Z. Oussadi A. (2012). Ecological characterization of the natural habitat of *Sulla* (Fabaceae) in Northeastern Algeria. Rev. D' Ecol. 67, 295–304. Available online at: http://hdl.handle.net/2042/55922

[B21] Lesniak J. Barton W. A. Nikolov D. B. (2002). Structural and functional characterization of the Pseudomonas hydroperoxide resistance protein Ohr. EMBO J. 21, 6649–6665. 10.1093/emboj/cdf670 12485986 PMC139091

[B22] Long S. R. (2001). Genes and signals in the *Rhizobium-legume* symbiosis. Plant Physiol. 125, 69–72. 10.1104/pp.125.1.69 11154299 PMC1539328

[B23] Marx H. Minogue C. E. Jayaraman D. Richards A. L. Kwiecien N. W. Sihapirani A. F.. (2016). A proteomic atlas of the legume *Medicago truncatula* and its nitrogen-fixing endosymbiont *Sinorhizobium meliloti*. A proteomic atlas of the legume *Medicago truncatula* and its nitrogen-fixing endosymbiont *Sinorhizobium meliloti*. Nat. Biotechnol. 34, 1198–1205 10.1038/nbt.3681 27748755 PMC8557956

[B24] Meneghetti F. Alberghini S. Tola E. Giacomini A. Ollero F. J. Squartini A. (1996). Presence of unique repeated insertion sequences in nodulation genes of *Rhizobium ‘hedysari.’* Plant Soil 186, 113–120. 10.1007/BF00035064

[B25] Muresu R. Sulas L. Polone E. Squartini A. (2005). PCR primers based on different portions of insertion elements can assist genetic relatedness studies, strain fingerprinting and species identification in rhizobia. FEMS Microbiol. Ecol. 54, 445–453. 10.1016/j.femsec.2005.05.008 16332341

[B26] Patschkowski T. Schluter A. Priefer U. B. (1996). *Rhizobium leguminosarum* bv viciae contains a second fnr/fixK-like gene and an unusual fixL homologue. Mol. Microbiol. 21, 267–280. 10.1046/j.1365-2958.1996.6321348.x 8858582

[B27] Remigi P. Zhu J. Young J. P. W. Masson-Boivin C. (2016). Symbiosis within symbiosis: evolving nitrogen-fixing legume symbionts. Trends Microbiol. 24, 63–75. 10.1016/j.tim.2015.10.007 26612499

[B28] Sato S. Nakamura Y. Kaneko T. Asamizu E. Kato T. Nakao M.. (2008). Genome structure of the legume, *Lotus japonicus*. DNA Res. 15, 227–239. 10.1093/dnares/dsn008 18511435 PMC2575887

[B29] Seemann T. (2014). Prokka: rapid prokaryotic genome annotation. Bioinformatics 30, 2068–2069. 10.1093/bioinformatics/btu153 24642063

[B30] Spaink H. P. Wijffelman C. A. Pees E. Okker R. J. H. Lugtenberg B. J. J. (1987). Rhizobium nodulation gene nodD as a determinant of host specificity. Nature 328, 337–340. 10.1038/328337a0

[B31] Squartini A. Dazzo F. B. Casella S. Nuti M. P. (1993). The root nodule symbiosis between *Rhizobium ‘hedysari’* and its drought-tolerant host *Hedysarum coronarium*. Symbiosis 15, 227–238.

[B32] Squartini A. Struffi P. Döring H. Selenska-Pobell S. Tola E. Giacomini A.. (2002). *Rhizobium sullae* sp. nov., (formerly '*Rhizobium hedysari'*), the root-nodule microsymbiont of *Hedysarum coronarium* L. Int. J. Syst. Evol. Microbiol. 52, 1267–1276. 10.1099/00207713-52-4-1267 12148639

[B33] Struffi P. Corich V. Giacomini A. Benguedouar A. Squartini A. Casella S.. (1988). Metabolic properties, stress tolerance and macromolecular profiles of rhizobia nodulating *Hedysarum coronarium*. J. Appl. Microbiol. 84, 81–89. 10.1046/j.1365-2672.1997.00318.x 15244061

[B34] Tola E. Henriquez-Sabà J. L. Polone E. Dazzo F. B. Concheri G. Casella S. (2009). Shovel roots: a unique stress-avoiding developmental strategy of the legume plant *Hedysarum coronarium* L. Plant Soil 322, 25–37. 10.1007/s11104-008-9861-4

[B35] Tolin S. Arrigoni G. Moscatiello R. Masi A. Navazio L. Sablok G.. (2013). Quantitative analysis of the naringenin-inducible proteome in *Rhizobium leguminosarum* by isobaric tagging and mass spectrometry. Proteomics 13, 1961–1972. 10.1002/pmic.201200472 23580418

[B36] Trotter V. Vinella D. Loiseau L. De Choudens S. O. Fontecave M. Barras F. (2009). The CsdA cysteine desulphurase promotes Fe/S biogenesis by recruiting Suf components and participates to a new sulphur transfer pathway by recruiting CsdL (ex-YgdL), a ubiquitin-modifying-like protein. Mol. Microbiol. 74, 1527–1542. 10.1111/j.1365-2958.2009.06954.x 20054882

[B37] Wang D. Yang S. M. Tang F. Zhu H. Y. (2012). Symbiosis specificity in the legume - rhizobial mutualism. Cell. Microbiol. 14, 334–342. 10.1111/j.1462-5822.2011.01736.x 22168434

[B38] Ward N. Moreno-Hagelsieb G. (2014). Quickly finding orthologs as reciprocal best hits with BLAT, LAST, and UBLAST: How much do we miss? PLoS ONE 9:e101850. 10.1371/journal.pone.0101850 25013894 PMC4094424

[B39] Yates R. Howieson J. De Meyer S. E. Tian R. Seshadri R. Pati A.. (2015). High-quality permanent draft genome sequence of *Rhizobium sullae* strain WSM1592; a *Hedysarum coronarium* microsymbiont from Sassari, Italy. Stand. Genomic Sci. 10, 1–6. 10.1186/s40793-015-0020-2 26380632 PMC4572446

[B40] Young J. P. W. Crossman L. Johnston A. Thomson N. Ghazoui Z. Hull K.. (2006). The genome of *Rhizobium leguminosarum* has recognizable core and accessory components. Genome Biol. 7:R34. 10.1186/gb-2006-7-4-r34 16640791 PMC1557990

[B41] Young N. D. Debellé F. Oldroyd G. E. Geurts R. Cannon S. B. Udvardi M. K.. (2011). The *Medicago* genome provides insight into the evolution of rhizobial symbioses. Nature 480, 520–524. 10.1038/nature10625 22089132 PMC3272368

