# Supplementary material for: Draft Genome Sequence of the Nitrogen-Fixing Rhizobium sullae Type Strain IS123T Focusing on the Key Genes for Symbiosis with its Host Hedysarum coronarium L
Source: Front Microbiol. 2017 Jul 26;8:1348. doi: 10.3389/fmicb.2017.01348 (PMC5526965; doi:10.3389/fmicb.2017.01348)
Supplement: Supplementary file 1 [file DataSheet1.DOCX]

Supplementary Material S1

Draft genome sequence of the nitrogen-fixing Rhizobium sullae type strain IS123T overviewing key genes for the symbiosis with its host Hedysarum coronarium L.

**Gaurav Sablok^*^, Riccardo Rosselli, Torsten Seeman, Robin van Velzen, Elisa Polone, Alessio Giacomini, Nicola La Porta , Rene Geurts, Rosella Muresu, Andrea Squartini**

*** Correspondence:**: Gaurav Sablok (sablokg@gmail.com)


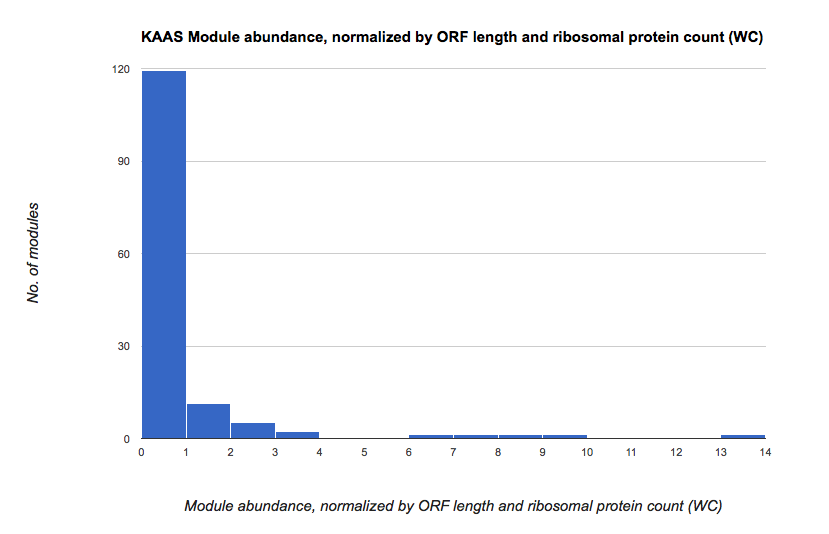


**Supplementary Figure 1.** KAAS (KEGG Automatic Annotation Server) Modules resulting from the mapping of the *Rhizobium sullae* IS123^T^ proteins in the Kyoto Encyclopedia of Genes and Genomes (KEGG) normalized by the ribosomal proteins counts.


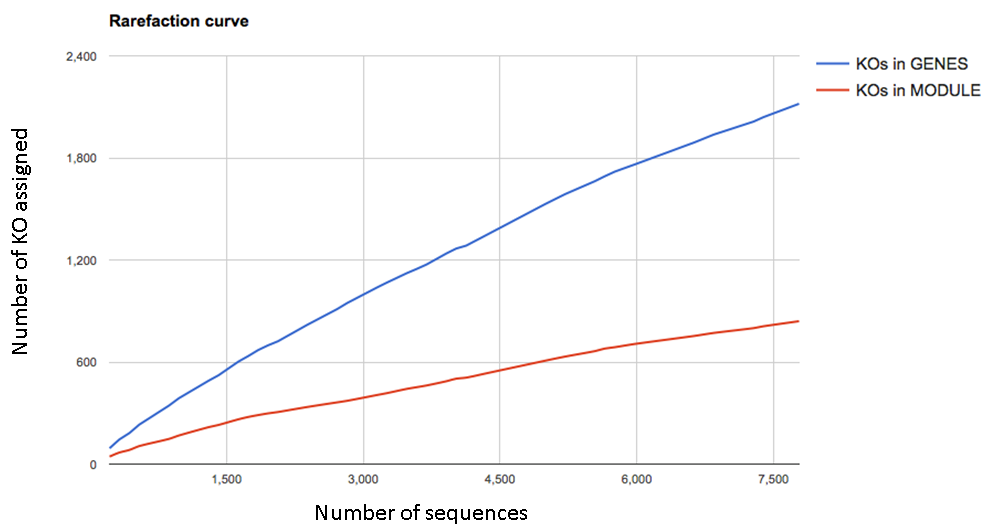
**Supplementary Figure 2. Rarefaction curves of** the [KO](http://www.genome.jp/kegg/ko.html) (KEGG Orthology) assignments mapped on either the genes or the modules.


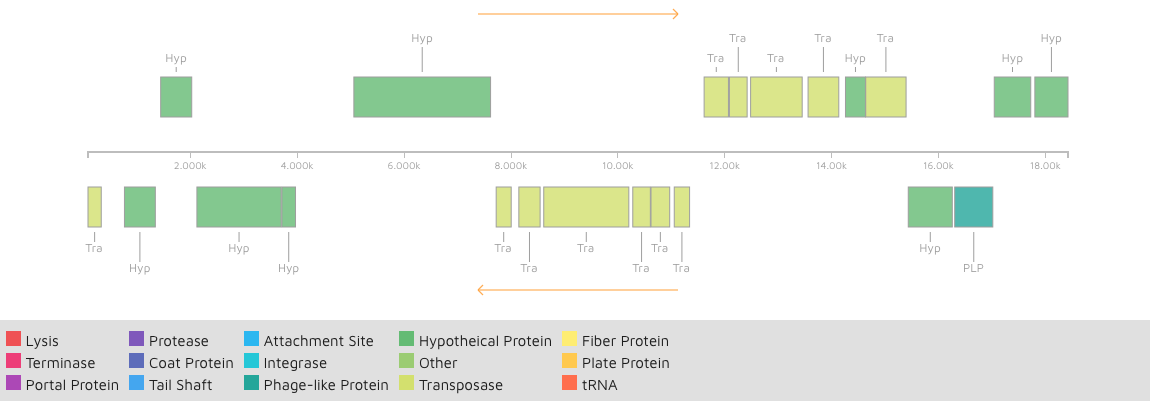


**Supplementary Figure 3.** Annotated map of the integrated prophage found in the genome of *R.sullae* IS123^T^.
